# Isothermal amplification of environmental DNA (eDNA) for direct field-based monitoring and laboratory confirmation of *Dreissena* sp.

**DOI:** 10.1371/journal.pone.0186462

**Published:** 2017-10-16

**Authors:** Maggie R. Williams, Robert D. Stedtfeld, Cathrine Engle, Paul Salach, Umama Fakher, Tiffany Stedtfeld, Erin Dreelin, R. Jan Stevenson, Jo Latimore, Syed A. Hashsham

**Affiliations:** 1 Department of Civil and Environmental Engineering, Michigan State University, East Lansing, Michigan, United States of America; 2 Department of Fisheries and Wildlife, Michigan State University, East Lansing, Michigan, United States of America; 3 Center for Water Sciences, Michigan State University, East Lansing, Michigan, United States of America; 4 Department of Integrative Biology, Michigan State University, East Lansing, Michigan, United States of America; 5 Center for Microbial Ecology, Michigan State University, East Lansing, Michigan, United States of America; University of Hyogo, JAPAN

## Abstract

Loop-mediated isothermal amplification (LAMP) of aquatic invasive species environmental DNA (AIS eDNA) was used for rapid, sensitive, and specific detection of *Dreissena* sp. relevant to the Great Lakes (USA) basin. The method was validated for two uses including i) direct amplification of eDNA using a hand filtration system and ii) confirmation of the results after DNA extraction using a conventional thermal cycler run at isothermal temperatures. Direct amplification eliminated the need for DNA extraction and purification and allowed detection of target invasive species in grab or concentrated surface water samples, containing both free DNA as well as larger cells and particulates, such as veligers, eggs, or seeds. The direct amplification method validation was conducted using *Dreissena polymorpha* and *Dreissena bugensis* and uses up to 1 L grab water samples for high target abundance (e.g., greater than 10 veligers (larval mussels) per L for *Dreissena* sp.) or 20 L samples concentrated through 35 μm nylon screens for low target abundance, at less than 10 veligers per liter water. Surface water concentrate samples were collected over a period of three years, mostly from inland lakes in Michigan with the help of a network of volunteers. Field samples collected from 318 surface water locations included i) filtered concentrate for direct amplification validation and ii) 1 L grab water sample for eDNA extraction and confirmation. Though the extraction-based protocol was more sensitive (resulting in more positive detections than direct amplification), direct amplification could be used for rapid screening, allowing for quicker action times. For samples collected between May and August, results of eDNA direct amplification were consistent with known presence/absence of selected invasive species. A cross-platform smartphone application was also developed to disseminate the analyzed results to volunteers. Field tests of the direct amplification protocol using a portable device (Gene-Z) showed the method could be used in the field to obtain results within one hr (from sample to result). Overall, the direct amplification has the potential to simplify the eDNA-based monitoring of multiple aquatic invasive species. Additional studies are warranted to establish quantitative correlation between eDNA copy number, veliger, biomass or organismal abundance in the field.

## Introduction

The use of environmental DNA (eDNA) for aquatic invasive species (AIS) detection has the potential to increase the likelihood of early detection [1] and enhance the probability of successful eradication [2]. Simplifying the analytical approach and decreasing the time-to-result is a key first step in developing rapid, field-deployable nucleic acid- based eDNA detection methods. Direct amplification, i.e., amplification without DNA extraction or purification, satisfies both these attributes. Elimination of DNA extraction and purification steps simplifies the process and may avoid the need for sample transport [3,4]. For detection of invasive species at very low abundance, sample concentration is often useful and necessary. However, sample concentration may also lead to simultaneous concentration of substrates inhibitory to Taq polymerases used in polymerase chain reaction (PCR)-based eDNA assays [5].

Isothermal amplification polymerases (such as *Bst* polymerase), have been found to be less impacted by the PCR inhibitors [6–8]. Compared to Taq polymerases, they have been shown to work significantly better even when crude lysates and whole cells are used as targets for amplification [3]. The loop-mediated isothermal amplification (LAMP) technique is one such isothermal approach (63°C) that utilizes *Bst* polymerase. LAMP could be well-suited for directly amplifying eDNA including cells, juveniles, eggs, or seeds, without extensive cell lysis and has been shown to directly amplify relatively unprocessed biological material such as cells, spores, and parasites [9–13]. Hence, direct isothermal amplification (i.e., amplification without carrying out DNA extraction and purification), combined with simpler field-deployable concentration approaches for samples containing much lower abundance of target species, have the potential to complement eDNA-based surveillance programs for invasive species [14].

To enhance the likelihood of detection, sample concentration (increasing the quantity of DNA or particles per unit volume) must be performed for low population abundances and is typically conducted ina laboratory either by filtration of 45 mL to 2 L water samples [15–18] through membranes of 0.45 to 10 μm pore size filters followed by eDNA extraction [15,19] or by eDNA precipitation [20]. Filtration is time consuming and often leads to filter clogging. However, it is possible to filter large volumes which may be needed at very low abundances [21,22] by using larger pore size (e.g., 10 to 60 μm [15]) filters and simultaneously collect sloughed tissues, veligers, juveniles, and fecal matter. In fact, filtration of large volumes is routine using plankton net tows to collect and concentrate microscopic organisms [23].

Overall, invasive species surveillance programs are currently hampered by the number of samples and the time required in getting them to the lab for processing. We hypothesize that by concentrating these cells using larger pore size filters in combination with direct amplification of eDNA in the field (both extracellular and present within these larger cells), we can increase likelihood of detection by providing a rapid methodology that could eliminate the need for complex sample processing. Furthermore, providing a laboratory-based confirmation of results could increase sensitivity and enhance the likelihood of detection. In this study, a direct eDNA amplification approach based on loop-mediated amplification was developed for the rapid detection of *Dreissena* sp. in the field. This methodology is further confirmed by isothermal amplification in the laboratory using eDNA extracted from 1 L samples. To test the effectiveness of this method, a total of 318 surface water samples were collected and analyzed. The direct amplification protocol was also validated in a pilot experiment using a field-deployable, real time isothermal amplification device (Gene-Z) to evaluate amplification from sample-to-result under field conditions. To our knowledge, this study represents the first attempt of using a direct amplification approach for eDNA detection and has the potential for rapid (under 90 min), field-based detection of invasive *Dreissena* sp.

## Materials and methods

### Loop-mediated isothermal amplification for *Dreissena* sp. detection

Loop-mediated isothermal amplification mixture consisted of 1X isothermal amplification buffer (New England BioLabs; Ipswich, MA), 1.4 mM each dNTP (Invitrogen; Carlsbad, CA), 0.8 M Betaine solution (Sigma-Aldrich; St. Louis, MO), 6 mM MgSO_4_ (New England Biolabs; Ipswich, MA), 6.4 U Bst Polymerase 2.0 WarmStart (New England Biolabs, Ipswich, MA), 1 μL primer mixture (described in the next section), 20 μM SYTO82 Orange Fluorescent Nucleic Acid Stain (ThermoFisher Scientific; Waltham, MA), 2.8 μL DNA extract, and PCR-grade water to a 10 μL total reaction volume [24]. Incubation for amplification was performed using a Chromo4 real-time thermal cycler (BioRad; Hercules, CA) located in a separate room (to eliminate contamination) using an isothermal protocol of incubation at 63°C for 60 min with fluorescence measured at one-minute intervals. Filtered pipets, sterile pipet tips, autoclaved tubes, and PCR-grade sterile water were also used. Negative and positive controls (n = 3 each) were run concurrently to ensure reagent quality and absence of contamination. Negative controls included PCR-grade water. Positive controls included DNA extracts containing *D*. *polymorpha* cytochrome c oxidase (CO1) target DNA. To prevent ambient contamination of amplicons after amplification, tubes were placed in zip lock bags and discarded in the separate room without ever opening the tubes. Benchtops were sterilized with 70% ethanol daily and 10% bleach weekly.

### Primer design for *Dreissena* sp.

Species-specific isothermal amplification primers were designed for the CO1 gene for *D*. *polymorpha* (Accession #: AF120663) and *D*. *bugensis* (Accession #: DQ840132; Table 1) using sequences obtained from GenBank [25]. One genus-specific sequence was also developed for *Dreissena* sp. using the 18S rRNA gene (Accession #: AF305702). Primer sets for each gene included six primers: loop forward (LF), loop backward (LB), forward (F3), backward (B3), forward inner primer (FIP), and backward inner primer (BIP). These were designed (Table 1) as per LAMP primer design requirements [24,26,27] using Primer Explorer V4 software and procured from Integrated DNA Technologies (Coralville, IA). The final primer mixture for the LAMP reaction contained 16 μM FIP and BIP, 8 μM LF and LB, and 2 μM F3 and B3.

**Table 1:** List of LAMP primers used in this study.

Species/Gene	Accession Number	Primer	Sequence (5’– 3’)
*Dreissena* sp./ 18S rRNA	AF305702	FIP	TGA AAG ATA CGT CGC CGG CGA ACT CGT GGT GAC TCT GGA C
		BIP	TGC CTA CCA TGG TGA TAA CGG GTG TCT CAT GCT CCC TCT CC
		LF	GTG CGA TCG GCA CAA AGT T
		LB	TAA CGG GGA ATC AGG GTT CG
		F3	GTT AGC CCA GAC CAA CGC
		B3	CTT CCT TGG ATG TGG TAG CC
*Dreissena polymorpha*/ cytochrome c oxidase (COI)	AF120663	FIP	AGA GAC AGG TAA AAC CCA AAA ACT AAT TGA TTG GTA CCA ATA ATA CTG AG
		BIP	ATT TTG TTC AGC TTT TAG GGA AGG AAA AAT CTA TCG CAG GGC C
		LF	CGA GGG AAA CCT ATA TCA GGA AGA
		LB	GGA TTC GGG GGT GGT TGA ACC
		F3	TAA TGG GGG GAT TCG GAA
		B3	GCT CCC CCA ATA TGA AGA G
*Dreissena bugensis/* cytochrome c oxidase (COI)	DQ840132	FIP	AAG AAG CTC CAC CGA TAT GAA GAG CCA CCG TTA TCC AGG ATT
		BIP	AGA ACA TGA GGA AAT ATA CGT GCC CAC CAA TAG AAG TAC AAA ACA AAG
		LF	ATG GCT GGC CCT GAA TGC C
		LB	GGG TGT CAT CAG TTT TAT CGG GT
		F3	ATT TGG TGG GGG TTG AAC
		B3	GGC TAA AAC AGG TAT TGC TAA

To establish analytical sensitivity, standard curves were prepared using 10X serial dilutions of target DNA in the range of 10 to 100,000 copies per reaction (using synthesized sequences). Species-specific LAMP assays were numerically evaluated using Basic Local Alignment Search Tool (BLAST [28]). Briefly, each primer sequence that was entered in BLAST was compared to sequences for mollusks and clams that are found in the same region (S1 File). Individual primers were evaluated for specificity by analyzing the following four BLAST parameters: max score, % query coverage, E value, and % identity. Primers of non-target species that have matching values to the target species are most likely to be non-specific. As LAMP utilizes 6 primers that target 8 regions, increased specificity to the target species is often observed as compared to qPCR, which only utilizes 2 primers [29]. Specificity was also determined experimentally by analyzing assays against related, non-target species.

### Validation of direct loop-mediated isothermal amplification of *D*. *polymorpha and D*. *bugensis* tissues and whole veligers

Amplification mixture for direct amplification followed the LAMP protocol described above except that 2.8 μL of extracted DNA was replaced by the same volume of crudely lysed water sample. For validation of the direct amplification procedure, samples of tissue from *D*. *polymorpha* and *D*. *bugensi*s were obtained from organisms found at Muskegon Lake (Muskegon Co., MI). Crude lysate was obtained by removing shells, crushing the entire remaining organism using a pestle, and vortexing for 1 min. Four mg of tissue (wet mass) was diluted with 1 mL of deionized water and serially diluted (10X; ranging from 1.12 μg to 1.12 ng), then 1 μL was added directly to the amplification reaction, with three replicates per dilution. Standard curves were generated for *D*. *polymorpha* and *D*. *bugensis* tissue mass using CO1 primers. This experiment was repeated thrice to account for run-to-run variation and average standard curves were generated for each (9 total replicates). Assay sensitivity was calculated based on the amplification of 9 replicates. The probability of detection was calculated for each dilution as the number of successful calls divided by the total number of replicates [30]. Best-fit straight trend lines for each data set were fitted, and the corresponding equations were used to determine the mass of target tissue. Using these standard curves, the mass present in each reaction was estimated for environmental samples, by comparing to the time to positivity (TTP) obtained.

To further validate the performance of direct amplification at much lower concentrations of mostly veligers and tissues, field samples were collected from Klinger Lake (St. Joseph Co., MI) using a plankton tow net (Wildco; Yulee, FL). Approximately 500 L of lake water was concentrated to a final volume of 500 mL and immediately transported to the laboratory for further analysis. The number of *D*. *polymorpha* veligers per mL of filtrate was counted under a microscope using a Sedgewick-Rafter counting cell (Wildco; Yulee, FL). Three serial dilutions of veligers were prepared in quadruplicate (0.09, 0.009, and 0.0009 veligers per μL) and subjected to: i) heat treatment at 95°C for 3 min, ii) pestle crushing, iii) heat treatment at 95°C for 3 min followed by pestle crushing, and iv) no treatment. Veligers were directly amplified without employing any DNA extraction procedure using *D*. *polymorpha* CO1 primers.

### Collection, processing, and analysis of surface water samples

**Fig 1:**
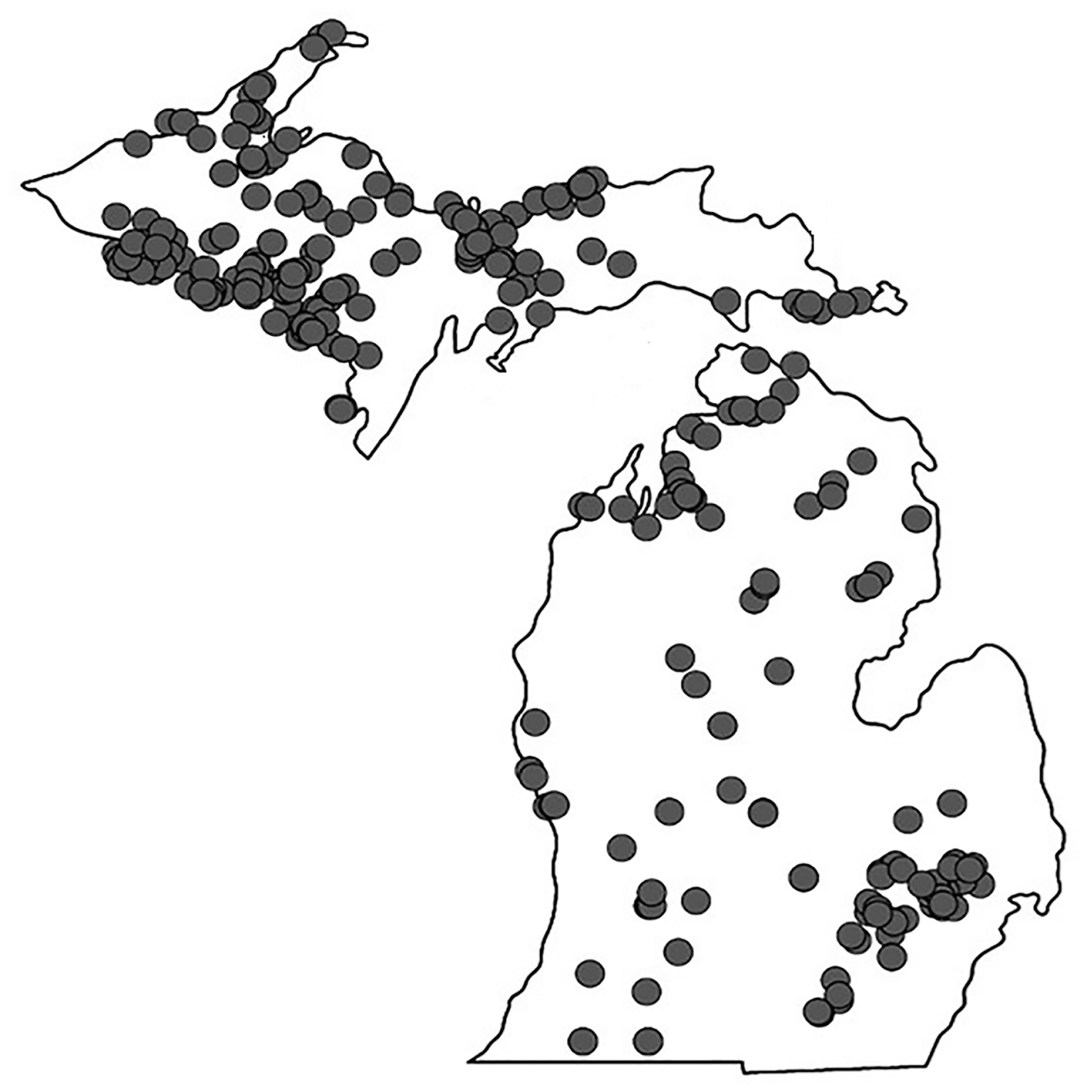
Location of 318 lake samples collected between November 2013 and August 2015.

Surface water samples were collected (a total of 318 samples; Fig 1) from lakes and streams located in Michigan and northern Wisconsin with assistance from over 100 volunteers (see Acknowledgements). Sampling kits provided to volunteers included: i) a filter funnel made by attaching a 35 μm mesh filter to a modified 1 L bottle with 35 μm mesh netting (Wildco, Yulee, FL), ii) conical tubes (50 mL), iii) a 1 L bottle for collection of grab water samples, and iv) instructions. Two sample types (a field-concentrated sample and an unconcentrated sample) were collected and sent to the laboratory for analysis.

The field- concentrated samples (n = 318) were obtained using a filter funnel with 35 μm mesh netting to achieve a 1000-fold concentration (20 L to 20 mL). Volunteers dipped the filter-funnel in the surface water 20 times to achieve concentration of 20 L. Following filtration, the 35 μm mesh filter and particulates were added to a conical tube containing 20 mL of the same surface water. Samples were then frozen (-20°C) immediately by volunteers for at least 12 h, then shipped to the laboratory via overnight shipping. Upon receipt samples were crudely lysed using a pestle, heated to 95°C for 3 min, and then promptly stored at -20°C until analysis to reduce chances for eDNA degradation [31].

To validate this sample concentration approach, samples were collected from two sites; one with a high population of *D*. *polymorpha* and another with a low population. At each location, two water samples were collected including an unfiltered water and concentrated water from the filter funnel (for 1000-fold concentration of AIS eDNA). To capture a high population abundance scenario (where there is a known infestation with peak reproduction seasons), samples were collected from Klinger Lake (St. Joseph Co., MI) in mid-June when high population densities have been previously observed.

To test how crucial the date/time of year of sample collection was for sensitivity, a selection of collected samples was obtained from the same location, but at multiple time points during the year. Concentrated samples (from 20 L to 20 mL) were collected from selected Michigan inland lakes including Klinger Lake (St. Joseph Co., MI), Au Train Lake (Alger Co., MI), Antoine Lake (Dickinson Co., MI), and Higgins Lake (Roscommon Co., MI).

A total of 174, 1 L grab un-concentrated surface water samples were also collected by volunteers to compare extracted DNA results with direct amplification. These were collected first by volunteers to ensure no contamination by the field- concentrated samples and also frozen immediately for at least 12 h before shipping overnight to the laboratory. Once received, surface water was filtered through 0.45 μm pore size filters (Millipore; Billerica, MA). DNA was then extracted using PowerWater DNA Isolation Kit (MoBio; Carlsbad, CA) following manufacturer’s protocols. Total DNA was quantified using Qubit 2.0 fluorometer with the Qubit dsDNA HS Assay Kit (ThermoFisher Scientific; Waltham, MA).

### Volunteer training

A smartphone application, termed “eDNA” was developed to train volunteers in sample collection and disseminate results. A video detailing the sample collection protocol was included as part of the application. In the documentation provided with the sample collection kit, particular emphasis was placed on sample handling and prevention of sample cross-contamination. Though equipment was pre-sterilized, volunteers were instructed to avoid sample to sample contamination and wash equipment thoroughly with a 10% bleach solution if contamination is suspected. Furthermore, samples collected at the beginning of this study by volunteers we collected in parallel with scientists to ensure similar results. The protocols provided emphasized that the sterilized sample collection bottles must only be opened once at the sampling location. To prevent DNA degradation within the collected sample, samples were frozen at -20°C within 4 h. Samples were then stored for at least 12 h until shipping to the laboratory for further processing and analysis. Samples were sent to the laboratory via overnight shipping and were typically still frozen upon arrival.

### Pilot tests using Gene-Z for rapid, field-based *Dreissena* sp. detection

Field tests of a portable gene analyzer (Gene-Z) were conducted at two locations: Klinger Lake (St. Joseph Co., MI) in June 2014 and Muskegon Lake (Muskegon Co., MI) in August 2015. Briefly, Gene-Z is a battery-operated, handheld gene analyzer that utilizes isothermal amplification and microfluidic cards capable of analyzing 64 isothermal reactions simultaneously [32]. The disposable cards are manufactured as previously described using a 40 W CO_2_ laser [7] and prior to field use, primer sets were dispensed into the reaction wells of each chip, dried, and stored at -20°C. At Klinger Lake, water samples were first collected using a hand filter and concentrated 1000-fold (from 20 L to 20 mL). At Muskegon Lake, water samples were collected without concentration step. Samples were then pipetted into a microfluidic chip which automatically distributes the samples into 64-wells using an airlock mechanism [3], then sealed with an optically transparent tape and inserted into Gene-Z device. The device was operated at an isothermal temperature of 63°C, with fluorescence measured every 15 seconds for each well. Fluorescence signals were tracked using an iPod touch, which also operated the device. Upon completion of the run, data was emailed from the iPod touch to a PC for further analysis in Excel.

### Data and statistical analysis

In all experiments, the following statistical analysis process was used. First, using raw fluorescence data, signal to noise ratio (SNR) at time t was calculated as the raw fluorescence minus the median background divided by the standard deviation of the average background signal. The TTP (the time at which the reaction is first positive) was calculated as the time when SNR crossed a threshold of ten [6]. All amplification reactions were performed in triplicate or higher. Based on positive amplification at the lowest copy numbers (1 target copy per well), a TTP of 50 min was selected as a cut-off for positive amplification. As stated earlier, the lower limit of detection for the assays was defined as the copy number at which at least 2 out of 3 replicates were positive [7]. The limit of quantification required at 3 out of 3 replicates (or a 95% detection level as is recommended [33]) to establish a standard deviation. Environmental samples were considered positive for the target of interest if positive signals were observed in at least two of the replicates [7] but were not used for quantification. A student's t-test was used to determine significant differences between two means using n-1 degrees of freedom and cutoff p-values of 0.05.

## Results and discussion

### Primer validation for analytical sensitivity and specificity with synthetic target gene DNA and extracted genomic DNA

From amplification reactions conducted with a dilution series of synthesized targets, the analytical sensitivity of the developed *D*. *polymorpha* and *D*. *bugensis* CO1 assays were calculated as 10,000 and 1,000 copies of target per reaction, respectively. For the 18S rRNA gene assay, the detection limit was 100 copies per reaction. In general, the primer set designed for the 18S rRNA gene was more sensitive than those designed for mitochondrial genes. Based on the known ideal LAMP primer parameters, this increased sensitivity for the primer set can, in part, be attributed to higher GC content [26] than the AT-rich CO1 genes. The mitochondrial CO1 genes have been reported to be more specific to the organism of interest, however, with more variability between species than other genes, making it ideally suited for eDNA detection [34,35]. These sensitivities were comparable with other studies [30,36]. It has also been suggested that more copies of mitochondrial genes are present in cells than other genes [37], which may allow primers that target CO1 to overcome GC content limitations.

**Fig 2:**
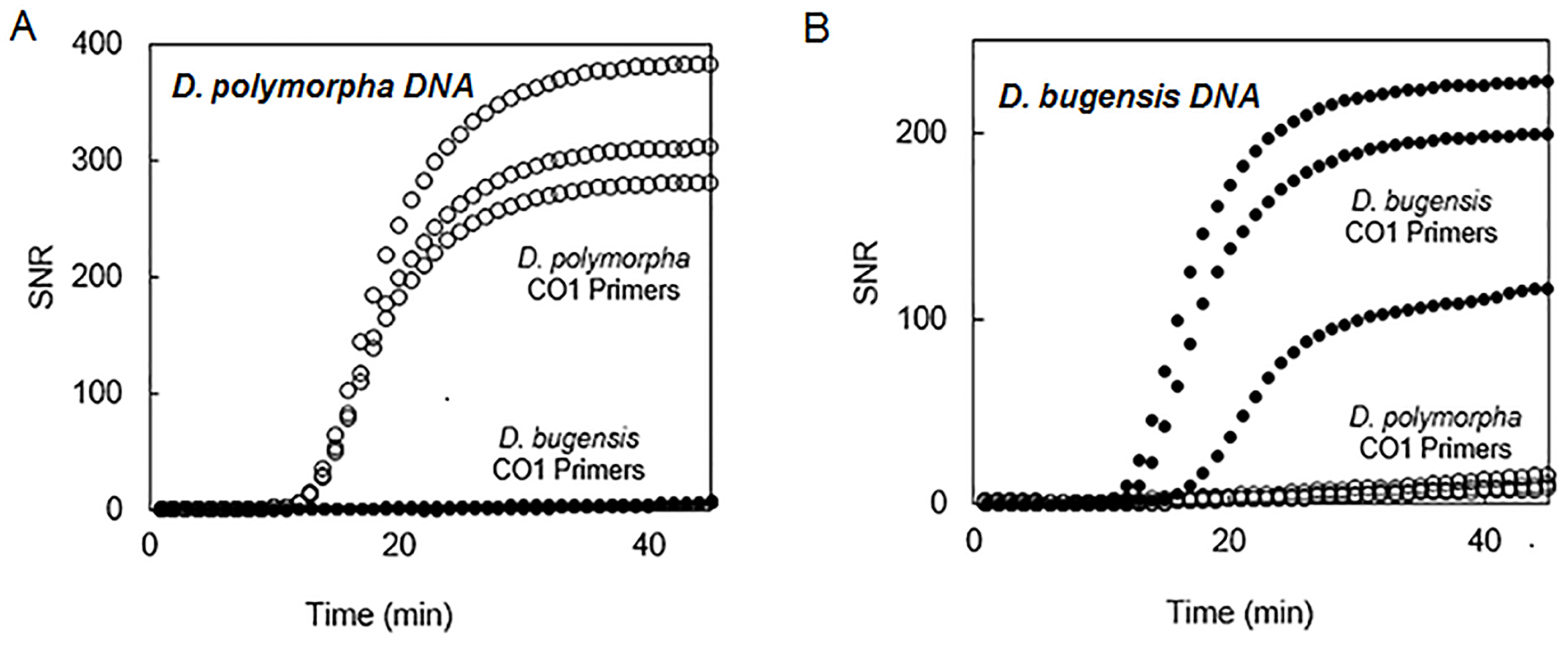
Specificity of assays validated with *D*. *polymorpha* and *D*. *bugensis*. *D*. *polymorpha* CO1 primers resulted in positive amplification only when *D*. *polymorpha* genomic DNA was present (A). Similarly *D*. *bugensis* CO1 primers gave amplification product only when *D*. *bugensis* genomic DNA was present (B).

In specificity assays using extracted genomic DNA from the two closely related *Dreissena* sp. and other mussels expected in MI lake waters, the primers specific to *D*. *polymorpha* only amplified *D*. *polymorpha* extracted DNA (TTP = 13 ± 0 min.; Fig 2A). Similarly, the primers designed to be specific to *D*. *bugensis* CO1 gene only gave amplification product only from *D*. *bugensis* extracted DNA (TTP = 14.3 ± 2.3 min.; Fig 2B). Primers for *Dreissena* sp. 18S rRNA gene successfully amplified DNA from both *D*. *polymorpha* and *D*. *bugensis*. Species-specific *Dreissena* sp. CO1 primers were also determined to be specific when tested experimentally against *Sphaerium* sp., *Viviparus* sp., and *Corbicula fluminea* (S1 Fig).

### Validation of primers for direct amplification from tissues and veligers

Using a dilution series prepared in the range of 0.1 ng/μL to 10 μg/μL of ground *D*. *polymorpha* and *D*. *bugensis* tissue samples, the sensitivity of the direct amplification of tissue was obtained. The detection limit was 0.01 μg tissue per reaction for the *D*. *polymorpha* CO1 gene assay, 0.001 μg tissue per reaction for the *D*. *bugensis* CO1 gene assay, and 0.0001 μg tissue per reaction for *Dreissena* sp. 18S rRNA gene assay (Table 2a). For field applicability, the likelihood of detection at a given tissue concentration was also calculated based on the number of positive reactions per set of 9 replicates (3 replicates each across 3 separate runs). For *D*. *polymorpha* CO1 primer sets, the likelihood of detection at 0.112 μg per reaction was 0.89 with 8 of the nine replicates yielding positive amplification. At 0.0112 μg per reaction and below none of the replicates amplified indicating that the likelihood of detection was close to zero. For *D*. *bugensis* likelihood of detection at 0.112 μg per reaction was 0.56 with 5 out of 9 replicates yielding positive results, and at 0.0112 μg per reaction, it was 0.375 with 3 out of 9 replicates with positive amplification. As this is first work investigating direct amplification of biomass for eDNA detection, we were not able to directly compare biomass sensitivities (0.000112–0.0112 μg tissue per reaction and 0.0009 veligers per reaction) to other studies, though other studies have linked eDNA results to organismal biomass [38]. It is possible that a small amount of extracellular DNA may also be detected, though DNA size is much smaller than 35 μm and thus may not be concentrated by this approach.

**Table 2:** Results obtained for different sample types including: *Dreissena polymorpha* tissues, *Dreissena bugensis* tissues, *D*. *polymorpha* veligers, 1000X concentrated water, and un-concentrated water. Information for each sample includes the location, the month and year of sample collection, sample processing, primers used, estimated target per reaction, and measured TTP.

Sample Type	Location	Month, Year Collected	Sample Processing	Primers	Target/Reaction	Av. TTP ± SD
***a*. *Direct amplification of Dreissena tissues***						
Tissue (*Dreissena* sp.)	Muskegon Lake (Muskegon Co., MI)	July, 2015	Heat Treatment*	*Dreissena* sp. 18S rRNA	11.12 μg	19.67 ± 0.71
					1.12 μg	20.78 ± 0.44
					0.112 μg	20.11 ± 1.05
					0.0112 μg	22.56 ± 2.55
					0.00112 μg	28.33 ± 7.70
					0.000112 μg	35.83 ± 10.13
Tissue (*Dreissena polymorpha*)	Muskegon Lake (Muskegon Co., MI)	July, 2015	Heat Treatment*	*Dreissena polymorpha* CO1	1111.2 μg	22.00 ± 0.00
					111.12 μg	22.67 ± 2.08
					11.12 μg	24.22 ± 2.64
					1.12 μg	26.00 ± 2.55
					0.112 μg	31.00 ± 4.32
Tissue (*Dreissena bugensis*)	Muskegon Lake (Muskegon Co., MI)	July, 2015	Heat Treatment*	*Dreissena bugensis* CO1	11.12 μg	27.13 ± 5.41
					1.12 μg	31.13 ± 5.14
					0.112 μg	40.20 ± 9.78
					0.0112 μg	41.00 ± 3.46
***b*. *Direct amplification of Dreissena polymorpha veligers***						
Veligers (*Dreissena polymorpha*)	Klinger Lake (St. Joseph Co., MI)	June, 2014	Heat Treatment*	*Dreissena polymorpha* CO1	0.09 veligers	33.67 ± 1.15
					0.009 veligers	32.33 ± 5.69
					0.0009 veligers	50.00^a^ ± N/A
			None		0.09 veligers	39.67 ± 1.53
					0.009 veligers	43.00 ^a^ ± N/A
					0.0009 veligers	ND**
***c*. *Effect of sample collection date on results***						
Lake water concentrate (1000X)	Klinger Lake (St. Joseph Co., MI)	Oct., 2013	Heat Treatment*	*Dreissena polymorpha* CO1	N/A	ND**
		May, 2014				ND**
		June, 2014				28.67 ± 6.35
Lake water concentrate (1000X)	Au Train Lake (Alger Co., MI)	Nov., 2013	Heat Treatment*	*Dreissena polymorpha* CO1	N/A	ND**
		July, 2014				29.67 ± 1.53
		Aug., 2014				ND**
Lake water concentrate (1000X)	Antoine Lake (Dickinson Co., MI)	Nov., 2013	Heat Treatment*	*Dreissena polymorpha* CO1	N/A	ND**
		Nov., 2014				22.67 ± 1.15
Lake water concentrate (1000X)	Higgins Lake (Roscommon Co., MI)	Oct., 2013	Heat Treatment*	*Dreissena polymorpha* CO1	N/A	ND**
		July, 2014				32 ± 0.00
Lake water concentrate (1000X)	Gun Lake (Barry Co., MI)	Sept., 2013	Heat Treatment*	*Dreissena polymorpha* CO1	N/A	ND**
		Oct., 2013				ND**
		May, 2014				42.0 ± 5.20
		Aug., 2014				ND**
		June, 2015				32.0 ± 0.00

^a^Only 2 of 3 replicates amplified.

*Heat Treatment = 95°C for 3 min.

**ND = Not Detected

Although, the whole genome information for *D*. *polymorpha* is still evolving, estimates are in the range of 1.7 pg per genome [39]. The total number of genes present in *D*. *polymorpha* (or other less studied mussels) is not yet fully assessed but studies related to *D*. *polymorpha* transcriptomics are emerging [40]. Based on the information gathered about genomes size and an assumption of 10,000 genes per 1.7 pg of DNA and a DNA: tissue weight ratio of 0.1%, the lower limit of detection was approximately 10^4^ gene copies per reaction for *D*. *polymorpha* at 0.112 μg tissue per reaction for CO1 gene. Further dilutions will of course lead to ~1 gene copy per reaction which will not always be present in each reaction well.

Direct amplification was also evaluated for *D*. *polymorpha* veligers in samples collected from Klinger Lake (St. Joseph Co., MI). Amplification was successful for as low as 0.09 veligers in the concentrated sample per reaction (TTP = 39.67 ± 1.53 min; Table 2b), without any sample processing. For 0.009 veligers per reaction, only one of the three replicates was positive and at 0.0009 veligers per reaction, no amplification was observed. Heat treatment enhanced the limit of detection with three of six replicates amplifying (six replicates included three for the mixed samples and three for non-mixed samples) for 0.0009 veligers per reaction. Heat treatment also improved the likelihood of detection, particularly at 0.009 veligers per reaction. All three replicates were positive, as opposed to only one of three successfully amplifying for the non-heat-treated group. In general, differences between the heat-treated and control groups were statistically significant (p = 0.0019). The effect of cell crushing using a pestle was not statistically significant (p = 0.065).

### Validation of filtration approach for sample concentration in the field

**Fig 3:**
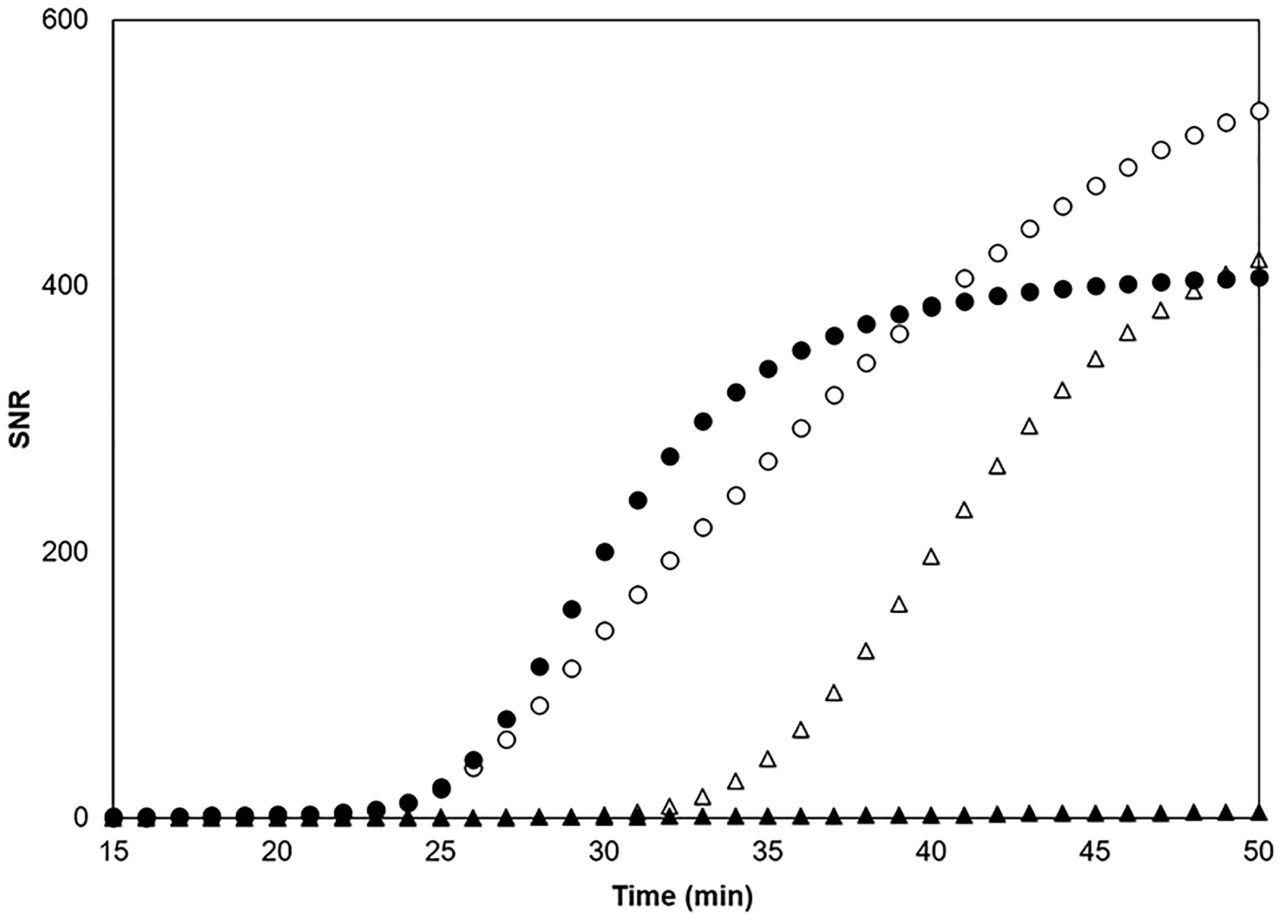
Direct amplification results for sample collection strategies including 1000X concentration (20 L hand-filtered to 20 mL) and un-concentrated water, at high initial population abundances (circles; open for concentrated and closed for un-concentrated) and low initial population abundances (triangles; open for concentrated and closed for un-concentrated). At high abundance, no change in TTP was observed between 1000X concentration and water-only. At low abundance, positive results were observed only after 1000X concentration.

To validate the filtration approach for sample concentration, results from concentrated samples were compared with un-concentrated surface water. At high abundances (samples collected at Klinger Lake in St. Joseph Co., MI in June) positive results were obtained from both sample types, suggesting that with large population abundances no sample concentration is required (concentrated sample TTP = 22.3 ± 3.2 min and un-concentrated sample TTP = 23.3 ± 1.53 min; Fig 3). Similarities in TTPs obtained can be attributed to the plateau in decreasing TTP observed in the standard curves of organismal biomass (Table 2a). For the lower population density case (where there is a known population but outside of reproduction peak season), samples were collected from Lake Lansing (Ingham Co., MI) in mid-November when veliger and tissue abundances are low. After concentrating the water sample by 1000-fold with the hand filter, positive amplification (concentrated sample TTP = 32.0 ± 3.0 min) was seen in all replicates. Without the concentration step, no amplification was observed in 60 min.

### Direct amplification of filtered surface water samples

**Fig 4:**
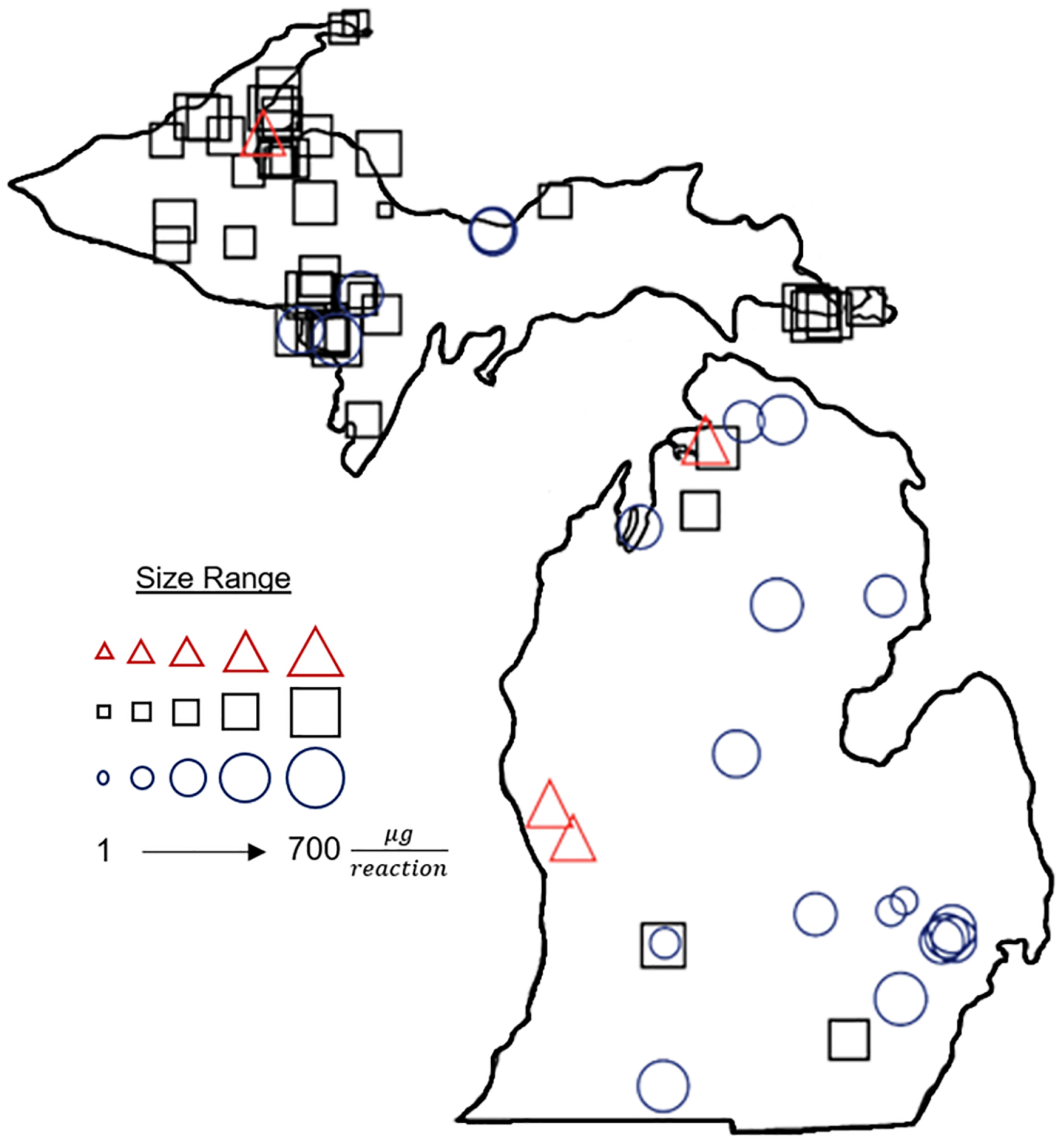
Results from direct amplification of environmental samples. Mass estimates for *D*. *polymorpha* CO1 (blue circles), *D*. *bugensis* CO1 (red triangles), and *Dreissena* sp. 18S rRNA (black squares). Larger shapes correspond to a high concentration tissue detected.

In general, detection of *D*. *polymorpha* was significantly widespread, with 27 positive detections throughout the state (Fig 4; S2 File). *D*. *bugensis* was only detected in 3 out of the 318 samples (Fig 4). A total of 168 out of 318 samples were also analyzed for *Dreissena* sp. and 59 samples were found positive. Increased observance of *Dreissena* sp. may be due to higher analytical sensitivity of the 18S rRNA gene primers compared to the species-specific primers.

Based on the results from the tissue mass presented in the above section, standard curves were generated for use in quantification of mass from field data using linear trendlines. While this is the first use of these standard curves for estimation of quantification of AIS tissue mass from field data for direct amplification, it is commonplace for quantification of DNA from Ct values obtained with qPCR [41,42] and has also been presented with LAMP for quantification of cells [43]. For *D*. *bugensis* CO1 gene primers, the equation used was y = −2.02 ln(x) + 32.571, where x is the mass and y is the TTP obtained. Similarly, for *D*. *polymorpha* CO1 and *Dreissena* sp. 18S rRNA, equations were y = −1.474 ln(x) +27.238 and y = −1.315 ln(x) +20.151, respectively. Theoretical mass at each location was also calculated based on the earlier presented linear trendline equations for tissue mass for *D*. *polymorpha* CO1, *Dreissena* sp. 18S rRNA and *D*. *bugensis* CO1. A visual representation of the mass values at each sampling location are also shown in Fig 4.

To obtain efficacy information about these results, known *D*. *polymorpha* infestation information was obtained from the United States Geological Survey (USGS) online database [44]. Of the total positive detections obtained from samples collected in the sampling period (May 2014 to August 2014 and May 2015 to August 2015; 171 out of 318 samples), 65.4% of which corresponded with reported infestations. For 15.4% of the total samples, previous *D*. *polymorpha* infestations were reported but not detected by the eDNA protocol, suggesting future studies could focus on the improvement of the detection limit or variability due to sampling locations.

For most lakes, direct amplification of was positive from approximately May to August during a given year. Time to positivity values obtained from the same locations by date is shown in Table 2c, using primers for *D*. *polymorpha* CO1. This may correspond with reproduction for *D*. *polymorpha*, which occurs when water temperatures exceed 12°C and would suggest that the number of veligers in the water column is much higher [45]. It also further confirms that the persistence of eDNA in the environment is important [46–48]. In the summer months, there is a potential for mixing from recreational activities which is at its peak [49]. Summer months are also the recommended time for completing *D*. *polymorpha* veliger surveys as well as other eDNA analysis methods [50]. This suggests that the implementation of the direct amplification method could complement these other approaches as they could be completed at similar times of the year. However, lakes (especially deep lakes) are typically stratified during warmer temperatures [51], which may complicate sample collection as there would not be complete mixing throughout the waterbody.

**Fig 5:**
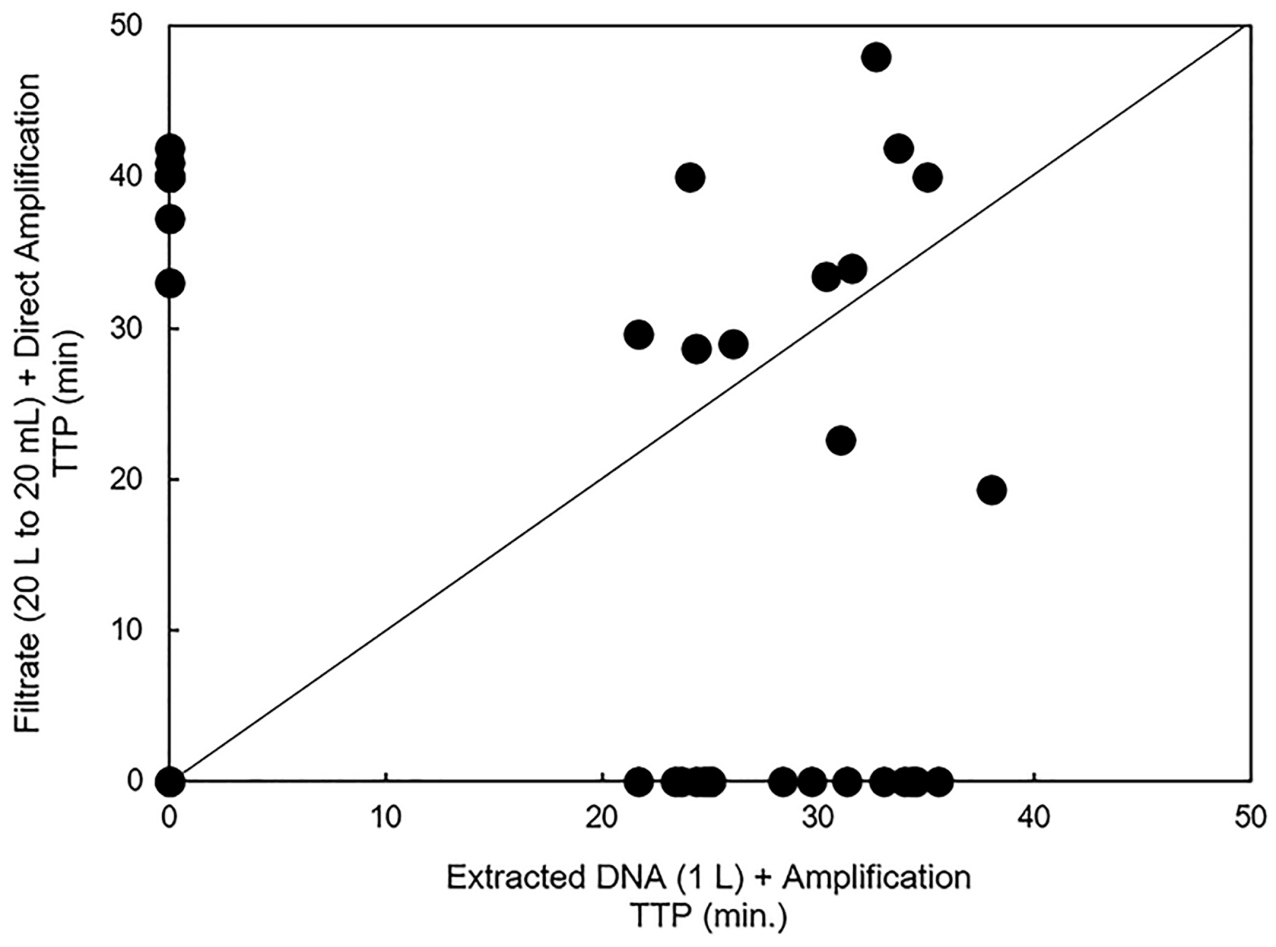
Comparison of results between the field-concentrated samples with direct amplification and the unconcentrated samples with DNA extraction and amplification. This 1:1 plot shows amplification results of the field-concentrated samples with direct amplification as compared to the results of 1 L unconcentrated samples following DNA extraction. Points along the y-axis only amplified with the field-concentrated samples and direct amplification while those along the x-axis only amplified with the unconcentrated method. Points in the center correspond to positive detections using both methods.

Of the 174 unconcentrated samples that were sent to the laboratory for DNA extraction and amplification analyzed and compared to their corresponding direct amplification sample, 11 were positive for *D*. *polymorpha* CO1 by both methods (Fig 5). A total of 5 positive results were obtained with direct amplification of filtrate samples and not with amplification of extracted DNA. A total 12 positive results were obtained with amplification of extracted DNA and not with direct amplification of filtrate.

### Results from pilot tests of Gene-Z for field-based detection of *Dreissena* sp.

In a pilot scale test at Klinger Lake (St. Joseph, MI) of a field-deployable device (Gene-Z) concentrated lake water was collected using the field-concentration approach. Once the filtrate was collected and crudely lysed as mentioned in the methods section, it was dispensed into the microfluidic cards and the card was sealed with optical film. Analyzing the concentrated lake water resulted in positive results for *D*. *polymorpha* using primers for the CO1 gene (TTP = 33.3 ± 3.8 min). When testing the un-concentrated water directly at Muskegon Lake (Muskegon, MI), positive detections were observed for *Dreissena* sp. (18S rRNA gene; TTP = 42.76 ± 8.8 min).

## Conclusions

The results obtained in this study through the collection and analysis of 318 samples supports that direct amplification may be useful for field monitoring of aquatic invasive species. While this is not the first study to analyze large numbers of samples for eDNA from aquatic invasive species [52] including *Dreissena* sp. [53], it is the first of its kind to analyze large numbers of samples for *Dreissena* sp. using LAMP. This highlights the advantages of a direct amplification-based eDNA approach, in that large numbers of samples are easily analyzed for dozens of species in a short time. Furthermore, through the laboratory-based confirmation of 1 L grab water samples, processed via filtration through a 0.45 μm filter following by DNA extraction, the likelihood of obtaining a positive result is significantly increased. The findings presented here show that extraction of DNA followed by LAMP may be slightly more sensitive than direct amplification and this is supported by the fold concentration of water that occurs with each (20 L– 20 mL for direct amplification; 1000-fold vs. 1 L– 50 μL for amplification following extraction; ~10,000 fold). The combination of both field-based direct amplification for rapid detection on- location combined with further laboratory confirmation would give more power to results obtained by increasing likelihood of detection overall, but also allowing rapid responses should positive results be obtained in the field.

Experiments conducted as part of this study show that the developed concentration technique and direct isothermal amplification combined with a field-deployable device could be used as a rapid warning tool to detect invasive species, with a total time required (from filtration to results) of about 90 min. When sample concentration is not needed due to high abundances, less than 30 min may be sufficient. By increasing the efficiency of AIS screening, often spread over a larger geographic area, it allows for more samples to be analyzed and thus enhances the likelihood of detection if a species is present. Appropriate location for such samples must obviously be decided based on field data. Moreover, the inclusion of volunteers reduces travel requirements and helps to educate and involve the public. Taken together, the procedure and programs developed here provide a useful tool for AIS detection. Data presented here describe the performance of an approach and platform for basin-wide surveillance using primers for *Dreissena* sp. Future studies should optimize the particulate concentration protocol for detection of other species (invasive or native), such as plant seeds.

## Supporting information

S1 Fig Results from the experimental specificity analysis. (TIF)

S1 File Results from the theoretical specificity analysis as conducted using BLAST. Individual primers were evaluated for specificity by analyzing max score, % query coverage, E value, and % identity.
(XLSX)

S2 File Direct amplification results. An excel datasheet is included with the results from direct amplification of all environmental samples.
(XLSX)
