# Supplementary figures and images for: Isothermal amplification of environmental DNA (eDNA) for direct field-based monitoring and laboratory confirmation of Dreissena sp
Source: PLoS One. 2017 Oct 16;12(10):e0186462. doi: 10.1371/journal.pone.0186462 (PMC5643059; doi:10.1371/journal.pone.0186462)

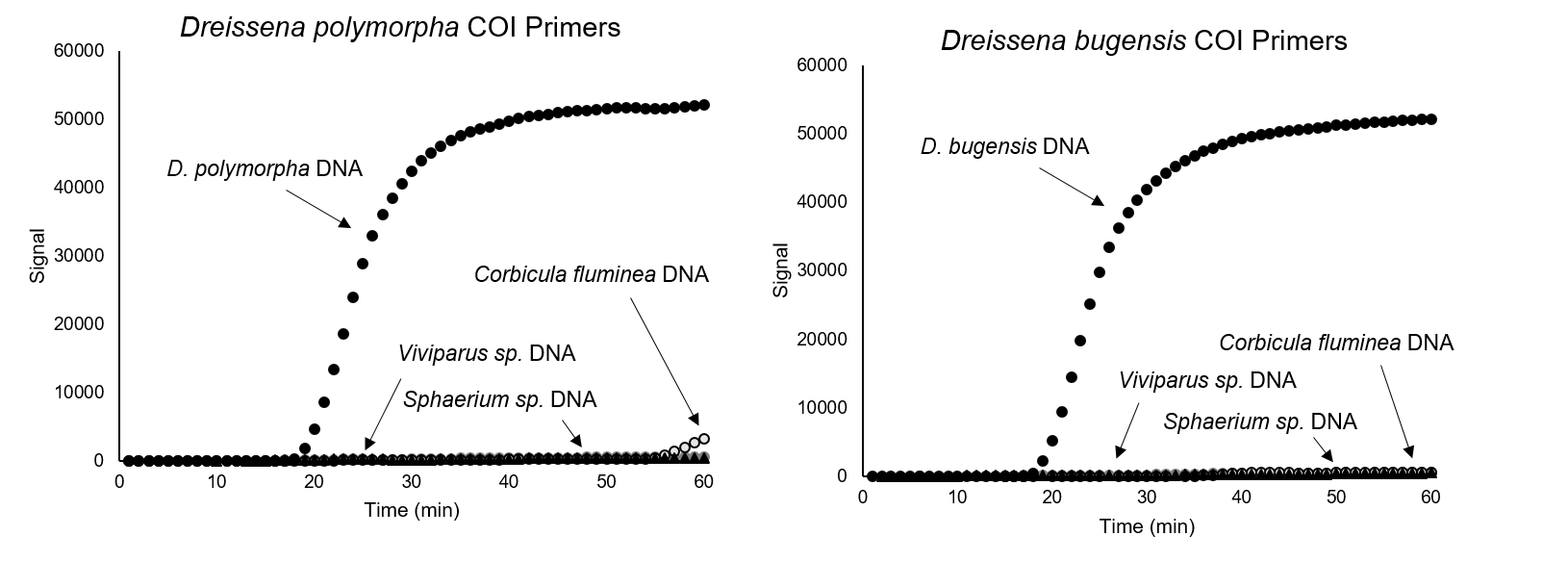

Supplement: S1 Fig — (TIF) [file pone.0186462.s001.tif]
